# Activated B cells steer fibroblasts towards a proinflammatory phenotype in giant cell arteritis

**DOI:** 10.1093/rheumatology/keag563

**Published:** 2026-10-19

**Authors:** Shuang Xu, William F Jiemy, Nanthicha Inrueangsri, YouBeen Ko, Wayel H Abdulahad, Ellen Verschoor, Yannick van Sleen, Jacoba C Graver, Kornelis S M van der Geest, Gwenny M Verstappen, Elisabeth Brouwer, Peter Heeringa, Annemieke M H Boots, Maria Sandovici

**Affiliations:** Department of Rheumatology and Clinical Immunology, University Medical Center Groningen, University of Groningen, Groningen, The Netherlands; Department of Rheumatology and Clinical Immunology, University Medical Center Groningen, University of Groningen, Groningen, The Netherlands; Department of Pathology and Medical Biology, University Medical Center Groningen, University of Groningen, Groningen, The Netherlands; Department of Rheumatology and Clinical Immunology, University Medical Center Groningen, University of Groningen, Groningen, The Netherlands; Department of Rheumatology and Clinical Immunology, University Medical Center Groningen, University of Groningen, Groningen, The Netherlands; Department of Pathology and Medical Biology, University Medical Center Groningen, University of Groningen, Groningen, The Netherlands; Department of Clinical Sciences, College of Medicine, University of Sharjah, Sharjah, UAE; Department of Rheumatology and Clinical Immunology, University Medical Center Groningen, University of Groningen, Groningen, The Netherlands; Department of Rheumatology and Clinical Immunology, University Medical Center Groningen, University of Groningen, Groningen, The Netherlands; Department of Rheumatology and Clinical Immunology, University Medical Center Groningen, University of Groningen, Groningen, The Netherlands; Department of Rheumatology and Clinical Immunology, University Medical Center Groningen, University of Groningen, Groningen, The Netherlands; Department of Rheumatology and Clinical Immunology, University Medical Center Groningen, University of Groningen, Groningen, The Netherlands; Department of Rheumatology and Clinical Immunology, University Medical Center Groningen, University of Groningen, Groningen, The Netherlands; Department of Pathology and Medical Biology, University Medical Center Groningen, University of Groningen, Groningen, The Netherlands; Department of Rheumatology and Clinical Immunology, University Medical Center Groningen, University of Groningen, Groningen, The Netherlands; Department of Rheumatology and Clinical Immunology, University Medical Center Groningen, University of Groningen, Groningen, The Netherlands

**Keywords:** giant cell arteritis, B-cells, fibroblasts, inflammation, therapy

## Abstract

**Objectives:**

Giant cell arteritis (GCA) is characterized by chronic large-vessel inflammation and remodelling. Within affected arteries, especially in aorta, organized B cell clusters are mainly localized in adventitia, where fibroblasts represent the dominant stromal population. This study aims to investigate fibroblast heterogeneity within these clusters and B cell–fibroblast interactions *in vitro* as possible contributors to disease chronicity in GCA.

**Methods:**

Fibroblast phenotypes in CD20^+^ B cell clusters in GCA and atherosclerotic aortic tissues were characterized using immunohistochemistry for fibroblast markers (CD90, podoplanin [PDPN], fibroblast activation protein [FAP], CD248, α-smooth muscle actin [α-SMA]) and cytokine expression (IL-6, GM-CSF, B-cell activating factor [BAFF]). *In vitro*, peripheral blood B cells from treatment-naïve GCA patients and healthy donors were stimulated and cocultured with human aortic adventitial fibroblasts (HAoAFs) using either direct cell coculture or transwell systems. To identify relevant molecular pathways for B cell–fibroblast interaction, HAoAFs were pre-treated with infliximab, tocilizumab, baricitinib or upadacitinib, followed by exposure to activated B cell conditioned medium. Protein secretion was assessed by ELISA, Luminex and Olink; mRNA expression was determined by qPCR.

**Results:**

CD90^+^PDPN^+^ immunofibroblasts mainly localized centrally, whereas CD90^+^FAP^+^ and CD90^+^CD248^+^ fibroblasts mostly located at the border of the clusters. Fibroblasts in or around clusters co-expressed IL-6, GM-CSF and BAFF. *In vitro*, HAoAFs cocultured with activated B cells or supernatants thereof upregulated IL-6, GM-CSF, PDPN and FAP mRNA, and actively secreted IL-6, GM-CSF and BAFF, while type I collagen expression was suppressed. Janus kinase–signal transducer and activator of transcription (JAK–STAT) inhibition abrogated these pro-inflammatory effects, whereas TNF-α and IL-6 receptor blockade did not.

**Conclusion:**

Activated B cells drive fibroblasts toward a pro-inflammatory phenotype, potentially sustaining chronic inflammation and disease progression in GCA. JAK–STAT signalling appears central to this effect, highlighting its therapeutic potency at the vascular level in GCA.

## Graphical abstract

**Graphical Abstract:**
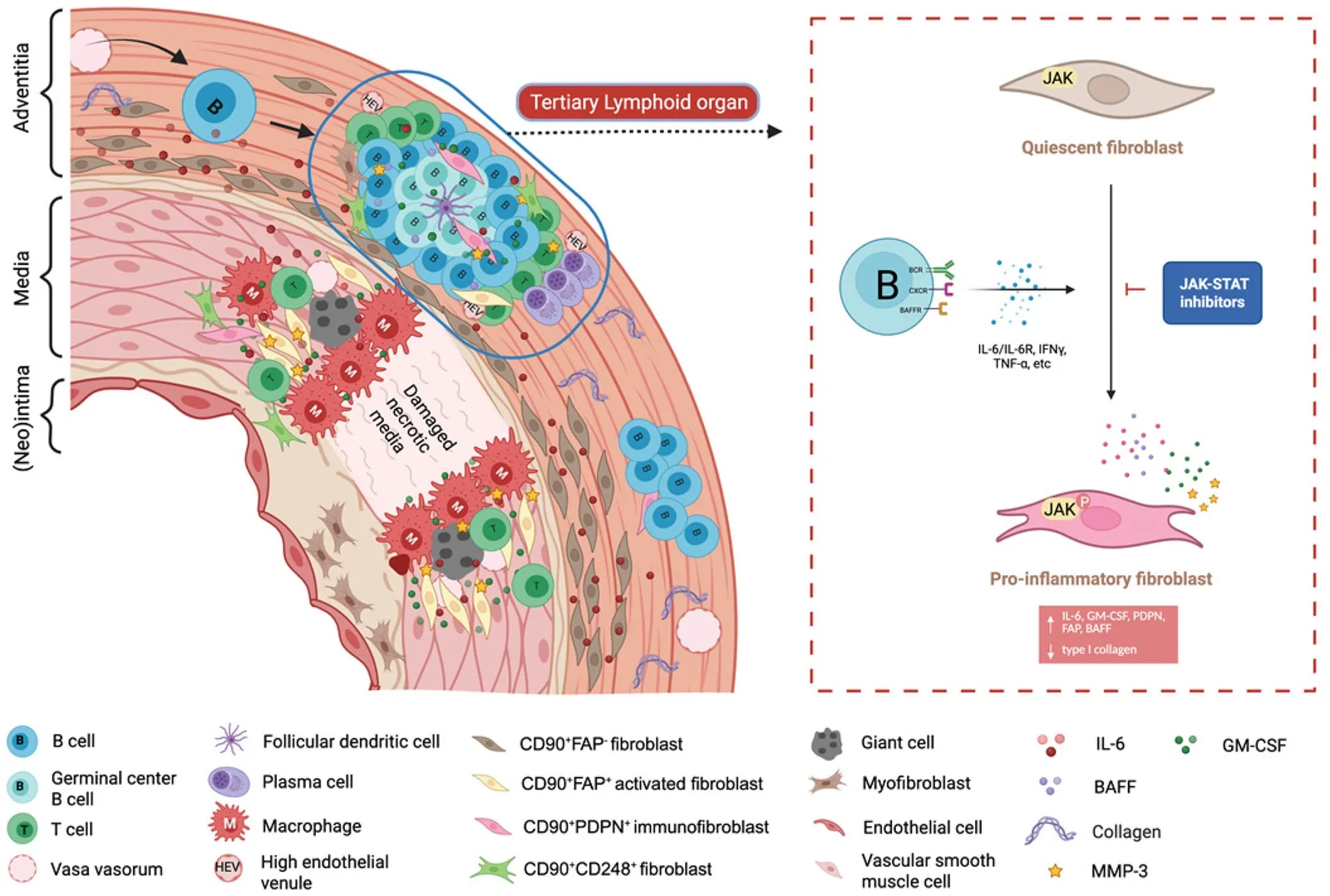
Proposed model of B cell effects on vascular adventitial fibroblasts in giant cell arteritis lesions. BAFF: B cell activating factor; FAP: fibroblast activation protein; GM-CSF: granulocyte–macrophage colony-stimulating factor; JAK–STAT: Janus kinase–signal transducer and activator of transcription; MMP-3: matrix metalloproteinase 3; PDPN: podoplanin. (Created with BioRender.com)

*Rheumatology* key messages

- Distinct spatial distribution of fibroblast subsets within and around adventitial B cell clusters in GCA aortitis.
- Activated B cells in GCA drive fibroblasts toward a pro-inflammatory phenotype, which can be attenuated by JAK–STAT inhibition.
- Targeting B cell and fibroblast crosstalk may present a promising therapeutic strategy for GCA.

## Introduction

Giant cell arteritis (GCA) is a granulomatous vasculitis of medium- and large-sized arteries, characterized by chronic inflammation and vascular remodelling, with or without polymyalgia rheumatica (PMR) [1]. Although several targeted immune therapies are being investigated for the treatment of GCA, only two, the IL-6 receptor blocker tocilizumab and the Janus kinase–signal transducer and activator of transcription (JAK–STAT) inhibitor upadacitinib, have thus far reached the clinical setting as glucocorticoid (GC)-sparing agents in GCA [2, 3]. While up to 60% of patients respond clinically to these targeted therapies, their impact on vascular pathology is still unclear. Insights into the cellular and molecular drivers of disease chronicity in GCA are instrumental for understanding the effects of current treatments on vasculitic lesions and for identifying new therapeutic targets.

While T cells and macrophages have long been considered the primary effector cells in GCA, accumulating evidence suggests that B cells and fibroblasts also play a role in disease persistence and tissue remodelling [4]. Although disease-specific antibodies have not been identified in GCA, B cells are present within vasculitic lesions, suggesting an antibody-independent role [5, 6]. Our group has previously reported massive infiltration of B cells in the adventitia of GCA-affected aorta tissues, outnumbering T cells [7]. B cells form arterial tertiary lymphoid organs (TLOs) in or near the adventitia, where they produce inflammatory mediators (e.g. IL-6, granulocyte-macrophage colony stimulating factor [GM-CSF], TNF-α and chemokines [CXCL9, CXCL13]) [5, 7]. Circulating B cells can also produce cytokines (IL-6, GM-CSF, TNF-α, IFN-γ and IL-10) [8, 9].

In recent years, fibroblasts have gained increasing attention as drivers of disease chronicity in autoimmune diseases. Fibroblasts are highly plastic cells that not only produce extracellular matrix (ECM) but also proliferate, secrete cytokines and growth factors, and orchestrate immune cell responses [10]. We recently described several fibroblast subtypes in arteries affected by GCA, including CD90^+^ fibroblast activation protein (FAP)^+^ activated fibroblasts, CD90^+^ podoplanin (PDPN)^+^ immunofibroblasts, CD90^+^ α-smooth muscle actin (α-SMA)^+^ myofibroblasts and CD90^+^CD248^+^ pro-fibrotic fibroblasts [11]. Adventitial and intimal CD90^+^ cells in temporal arteries from patients with GCA exhibit high levels of propyl 4-hydroxylase (P4H), α-SMA, myosin and FAP [12, 13].

The crosstalk of arterial fibroblasts with B cells in GCA is yet unexplored. This study aimed to characterize fibroblasts within B cell clusters in GCA aortitis, and explore B cell–fibroblast interaction through *in vitro* analyses.

## Methods

### Patients and controls

To study B cells and fibroblasts in tissues, aorta tissue samples from patients with histologically proven GCA (*n* = 9) and age- and sex-matched patients with atherosclerosis (*n* = 11) who underwent aortic aneurysm surgery were included. Patients’ clinical and laboratory data at the time of surgery were extracted from their medical records. All procedures were in compliance with the Declaration of Helsinki. Consent from the Internal Review Board and written patient consent were not required under the Dutch law for human medical research (WMO) since this was a retrospective, non-interventional study (UMCG Research Register number: 201800370). Patients were informed that obtained medical data could be used for research purposes in accordance with the General Data Protection Regulation of the European Union.

For the *in vitro* study, peripheral blood mononuclear cells (PBMC) from 12 treatment-naïve patients with GCA and 10 age- and sex-matched healthy donors were used for B cell isolation. All study participants provided written informed consent before inclusion (detailed information can be found in Supplementary Data S1). The study was approved by the institutional review board of the University Medical Centre Groningen (METc2010/222).

### Experimental methods

Paraffin-embedded aorta sections were used for immunohistochemistry (IHC) staining and OPAL multiplex immunofluorescence workflow. The detailed protocol is described in Supplementary Data S1, Supplementary Tables S1 and S2, and Supplementary Figs S1 and S5.

B cells were isolated from PBMCs through negative selection magnetic separation using the EasySep^TM^ Human B Cell Isolation Kit (STEMCELL Technologies, Cambridge, UK). After isolation, cells were stimulated, followed by coculturing with fibroblasts. Experimental procedures of PBMC isolation, B cell isolation, fibroblast culture and coculture can be found in Supplementary Data S1 and Supplementary Figs S2–S4.

mRNA levels of cocultured fibroblasts and protein secretion profiles of coculture systems were assessed by real-time quantitative PCR, ELISA, Luminex assay and Olink, respectively (Supplementary Data S1). Based on our previous work showing comparable cytokine production profiles of activated B cells from healthy controls and GCA patients under similar stimulation conditions, Luminex analyses were focused on GCA-derived cocultures to identify candidate mediators involved in B cell–fibroblast interactions [14]. For fibroblasts in the cell–cell contact model, the plate was washed three times and microscopically inspected to confirm the absence of non-adherent B cells prior to RNA isolation. In addition, CD20 mRNA expression was assessed by qPCR. The resulting *C*_T_ values were either very high (>38) or undetectable (>40), indicating minimal to no B cell contamination.

### Data analysis

To compare the IHC staining of different markers between GCA and atherosclerotic aortas, the Mann–Whitney *U*-test was performed, as the scores were not normally distributed. For the *in vitro* studies, Friedman’s test was used to compare mRNA levels in fibroblasts and protein production in supernatants between multiple groups. If overall significance was found, the Wilcoxon test was performed to compare the differences between two groups. The Mann–Whitney *U*-test was performed to compare the differences between the healthy control (HC) and GCA groups for a specific condition in the *in vitro* experiments. Statistical analyses were performed using GraphPad Prism 9.0 software (GraphPad Software, Boston, MA, USA), and *P* < 0.05 was considered statistically significant.

The downstream analyses of Olink data were performed in R version 4.4.3 using RStudio. Protein concentrations (pg/ml) were log2-transformed to reduce skewness and stabilize variance. Heatmaps were generated using the ComplexHeatmap package based on *Z*-score-scaled protein expression values. Hierarchical clustering was applied using Euclidean distance and complete linkage. Differential protein expression analysis was performed using the limma R package, where a linear model was fitted, and empirical Bayes statistics (eBayes) were applied to assess differential expression between groups. Multiple comparisons were adjusted using the Benjamini–Hochberg correction, and proteins with an adjusted *P*-value <0.05 were considered statistically significant. For the comparison of activated B cells between GCA and HC, a volcano plot was generated using uncorrected *P*-values to display the overall distribution of changes and highlight exploratory trends.

## Results

### Fibroblast subtypes show a specific location pattern in B cell clusters in aorta tissues

To evaluate the presence and distribution of fibroblasts in B cell clusters, we performed IHC and OPAL multiplex staining on aorta tissues of patients with GCA and with atherosclerosis as control group. Patient characteristics are presented in Table 1. Most B cells were observed in the adventitia and organized into clusters (Fig. 1A), where fibroblast markers were also identified (Supplementary Fig. S5). B cell clusters in the aortic adventitia showed abundant expression of CD90, PDPN and CD248. FAP expression was variable and generally limited in B cell clusters. α-SMA expression was mostly present around, but not in, the B cell clusters (Fig. 1B). Overall, fibroblast phenotypes in B cell clusters were comparable between GCA and atherosclerosis aorta tissues (data not shown). CD90^+^PDPN^+^ immunofibroblasts were mainly located in the centre of B cell clusters (Fig. 1C), whereas CD90^+^FAP^+^ activated fibroblasts and CD90^+^CD248^+^ fibroblasts were mostly located at the border of B cell clusters (Fig. 1D, E).

**Figure 1:**
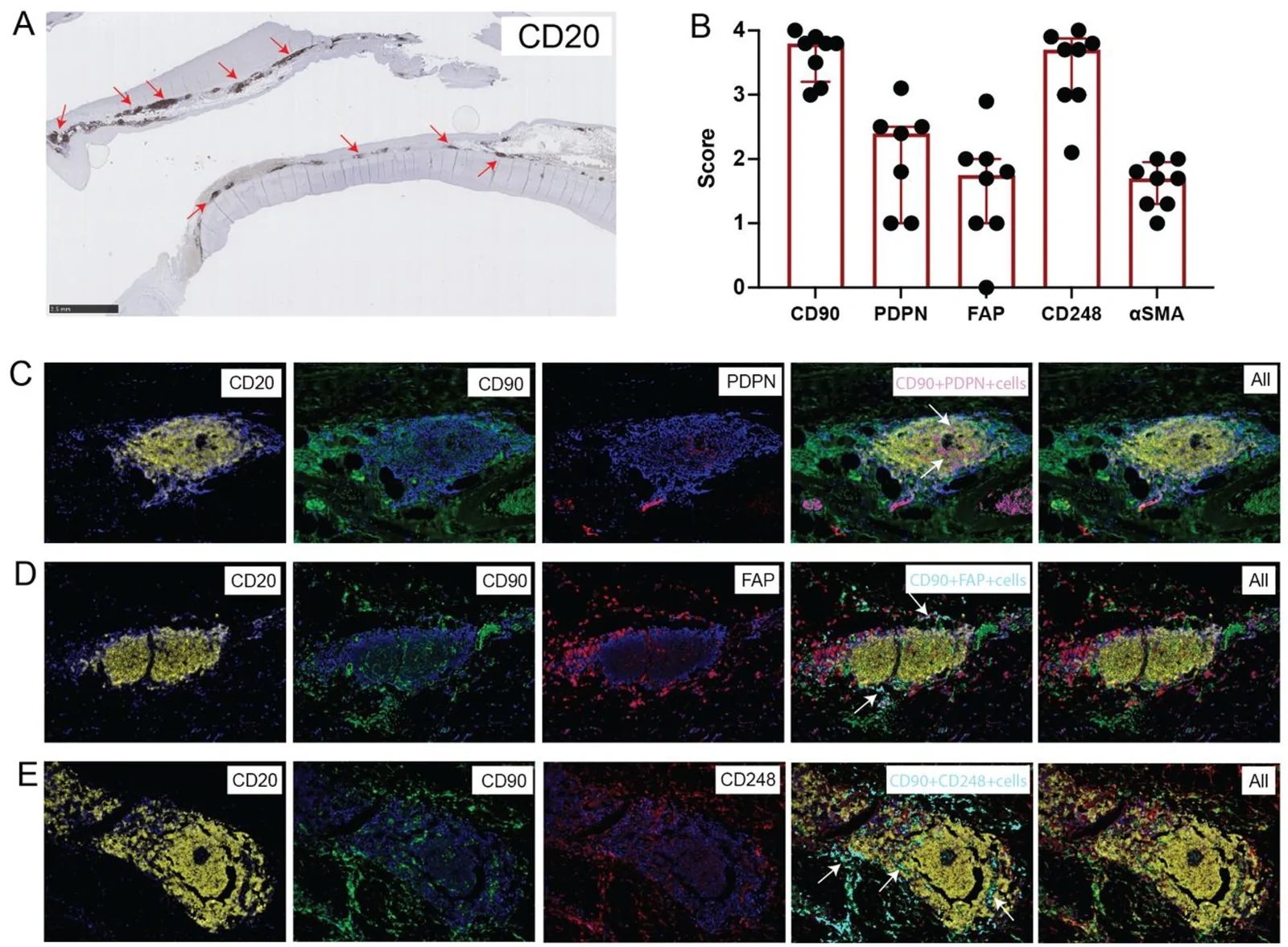
Detection of B cells and fibroblasts in aorta affected by giant cell arteritis (GCA). (**A**) Representative images of CD20 (pan B cell marker) expression in GCA aorta tissues. Red arrows indicate CD20 positive areas (brown staining). (**B**) Semi-quantitative analysis of fibroblast phenotype markers CD90 (pan-fibroblast), PDPN (immunofibroblasts), FAP (activated fibroblasts), CD248 (pro-fibrotic fibroblasts) and α-SMA (myofibroblasts) in B cell clusters in GCA aorta tissue. (**C–E**) Representative image of OPAL staining showing (as indicated by white arrows) CD90^+^PDPN^+^, CD90^+^FAP^+^ and CD90^+^CD248^+^ fibroblasts in and around B cell clusters. α-SMA: α-smooth muscle actin; FAP: fibroblast activation protein; PDPN: podoplanin

**Table 1:** Patient and healthy control characteristics.

Characteristic	Aorta		PBMC	
	GCA	AS	GCA	HC
Number of included individuals	9	11	12	10
Age, median (range), years	67 (55–79)	65 (59–78)	66 (46–83)	66 (62–76)
Sex, female, %	66.7	63.6	91.7	70
Individuals on treatment with glucocorticoids/immunosuppression	0	0	0	0
ESR, median (range), mm/h	24 (4–37)	17 (1–40)	50 (11–106)	7 (2–51)
Frequencies of positive ESR	4/7^a^	4/11	12/12	1/10
CRP, median (range), mg/l	7.5 (1.4–52)	9 (3–32)	41.5 (0.9–228)	<5
Frequencies of positive CRP	4/8^a^	4/11	10/12	0/10

a Two patients did not have ESR and one patient did not have CRP data before aorta operation. AS: atherosclerosis; CRP: C reactive protein; ESR: erythrocyte sedimentation rate; GCA: giant cell arteritis; HC: healthy controls; PBMC: peripheral blood mononuclear cell.

### Activated B cells promote a pro-inflammatory (*IL-6*, *GM-CSF*) and activated (*FAP*) mRNA profile of fibroblasts *in vitro*

To gain insight into the crosstalk between B cells and fibroblasts, B cells isolated from PBMCs of HCs and treatment-naïve GCA patients were used. Patient and HC characteristics are presented in Table 1. B cells were activated by incubation with CpG, PMA and CaI for 72 h, and then cocultured with human aortic adventitial fibroblasts (HAoAFs) for 24 h (Fig. 2A). The overall direction of fibroblast responses was largely comparable between HCs and GCA patients. Activated B cells from both GCA and HCs induced IL-6 expression in fibroblasts, although the magnitude appeared greater in GCA. Increased GM-CSF expression was primarily observed in fibroblasts cocultured with activated B cells from GCA patients. *B-cell activating factor* (*BAFF*) mRNA expression in fibroblasts was upregulated only by activated B cells from GCA patients through direct cell–cell contact, whereas *PDPN* upregulation was observed exclusively upon direct contact with activated B cells from HCs. *FAP* mRNA was upregulated in both models with no difference between GCA and HC groups. In contrast, *α-SMA* mRNA expression (Fig. 2B and Supplementary Fig. S6A) was not modulated by activated B cells. Furthermore, mRNA expression of *CXCL9*, *CXCL13* and *MMP-9* was not detectable in fibroblasts of any group (data not shown). These findings indicate that activated B cells can modulate fibroblast inflammatory and stromal gene expression, with largely comparable outcomes between the transwell and contact-dependent coculture systems, and that activated B cells from GCA have a higher potency to induce *IL-6*, *GM-CSF* and *BAFF* mRNA expression. Exploratory correlation analyses between ESR/CRP levels from GCA patients and mRNA expression profiles from cocultured fibroblasts showed that *FAP* mRNA levels in the transwell model correlated significantly with CRP levels (Fig. 2C and Supplementary Fig. S7A).

**Figure 2:**
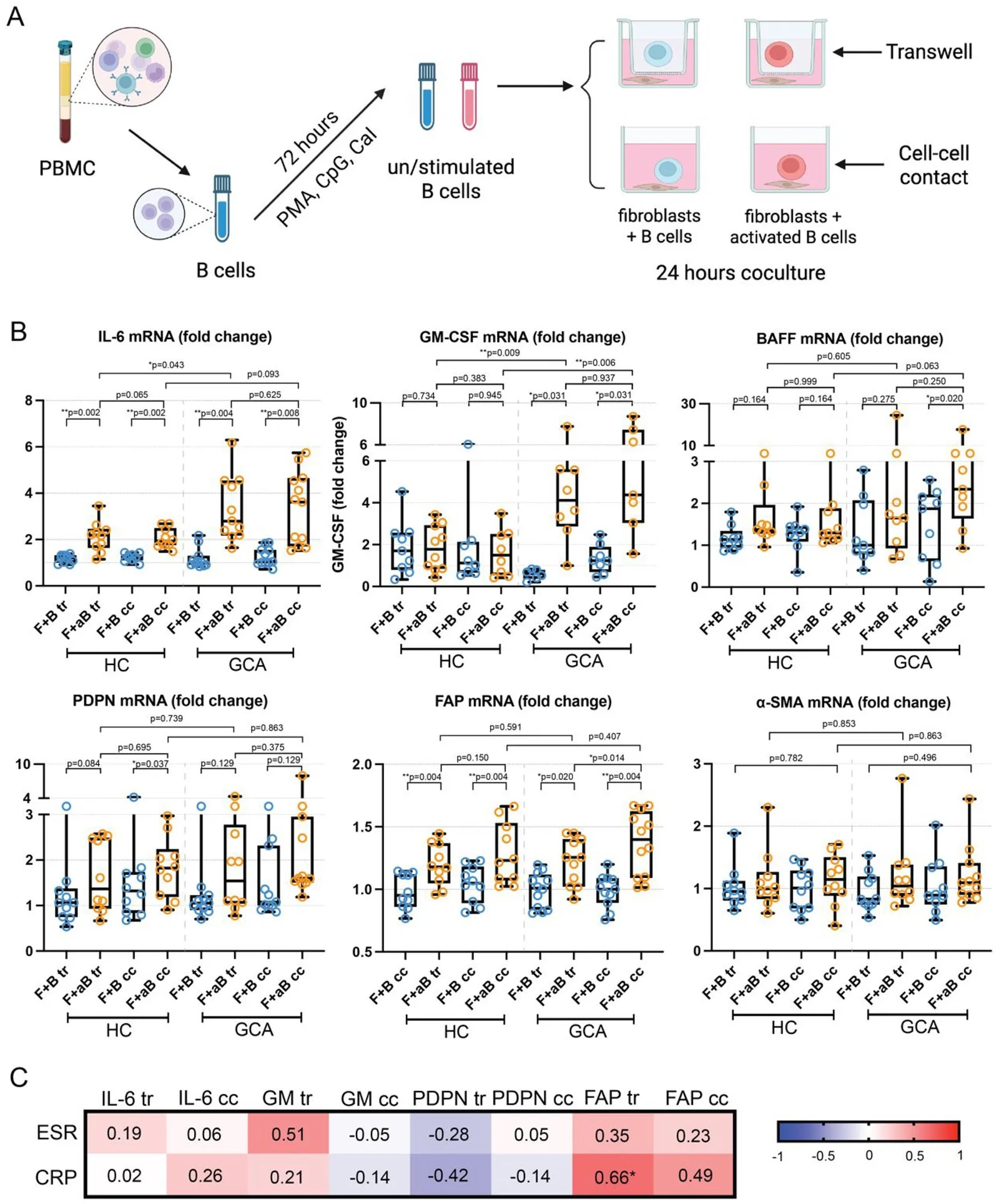
Effects of coculture with activated B cells on gene expression of inflammatory-, B cell survival- and fibroblast phenotype-associated markers on human aortic adventitial fibroblasts. (**A**) Schematic overview of the study design. (**B**) mRNA expression of *IL-6*, granulocyte-macrophages colony stimulating factor (*GM-CSF*), B cell activating factor (*BAFF*) podoplanin (*PDPN*), fibroblast activation protein α (*FAP*) and α-smooth muscle actin (*α-SMA*) in fibroblasts in different coculture systems. Fold change indicates comparison with the control group (fibroblast culture only). (**C**) Correlation of erythrocytes sedimentation rate (ESR) and C-reactive protein (CRP) with mRNA data. *P < 0.05, **P < 0.01; ns, not significant. aB: activated B cells; B: B cells; cc: cell–cell contact; CaI: calcium ionophore; CpG: CpG oligodeoxynucleotides (ODN) 2006; F: fibroblasts; BAFF: B-cell activating factor; GCA: giant cell arteritis; HC: healthy controls; PMA: phorbol 12-myristate 13-acetate; tr: transwell

### Activated B cells enhance pro-inflammatory cytokine secretion and suppress collagen production in fibroblasts *in vitro*

Next, we measured the protein levels of soluble factors related to inflammation (IL-6, GM-CSF, TNF-α, IFN-γ, CXCL8), B cell survival/activation (IL-6, BAFF), and damage/remodelling (matrix metalloproteinase [MMP]-3, pro-collagen I) in the supernatants of GCA B cell/fibroblast cocultures. The supernatant of fibroblasts cultured with activated B cells from GCA contained higher IL-6 protein levels under conditions of direct cell–cell contact than in the transwell coculture system (Fig. 3A). The supernatant of fibroblasts cultured alone or fibroblasts cocultured with activated B cells showed higher IL-6 protein levels compared with activated B cells alone (Supplementary Fig. S7B), which aligns with observations in GCA-affected aorta adventitia, where CD90 positive cells overlapped with ∼80% of the IL-6 positive area (Fig. 3C). Furthermore, the secretion of GM-CSF and BAFF was higher in the cell–cell contact compared with transwell system (Fig. 3B) while immunohistochemical analysis of GCA-affected aortic adventitia revealed expression of GM-CSF and BAFF by both B cells and fibroblasts (Fig. 3C). Notably, the secretion of pro-collagen Iα1 by fibroblasts was downregulated in the presence of activated B cells from GCA patients in both transwell and coculture systems, whereas this effect was observed only upon direct cell–cell contact with activated B cells from HCs (Fig. 3A). Accordingly, limited collagen deposition was observed in B cell clusters in GCA-affected aorta tissues (Fig. 3D). No significant differences were observed in the protein levels of CXCL8, MMP-3, TNF-α and IFN-γ in either system (Supplementary Fig. S7C). Collectively, these findings indicate that activated B cells modulate fibroblast function by enhancing the secretion of pro-inflammatory cytokines and survival factors while concurrently suppressing ECM production.

**Figure 3:**
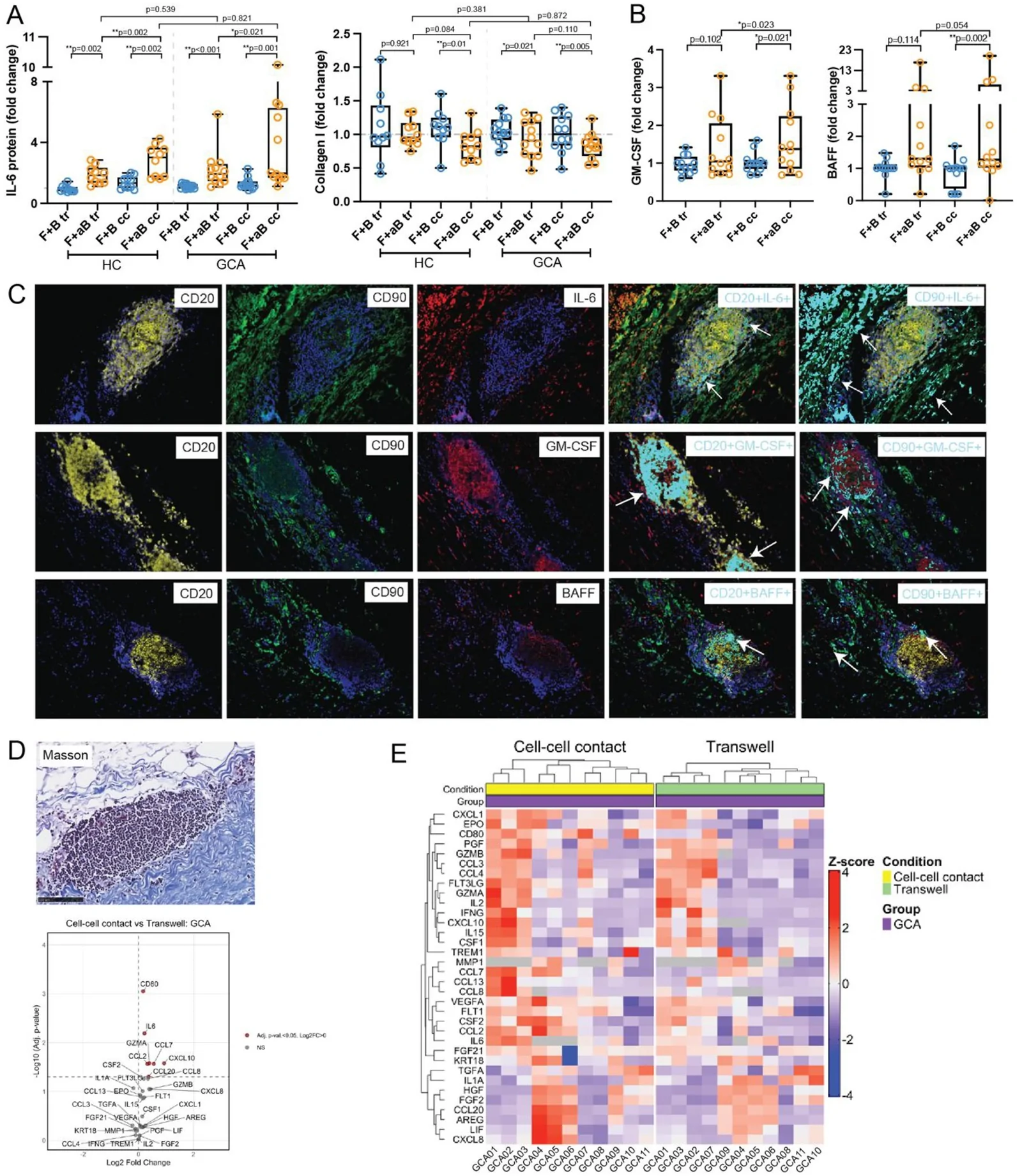
Protein secretion in the *in vitro* human aortic adventitial fibroblasts (HAoAF)–B cell coculture models. (**A**) Secretion of IL-6 protein and pro-collagen Iα1 (ELISA) in the coculture systems from both healthy controls (HC) and patients with giant cell arteritis (GCA). (**B**) Production of granulocyte–macrophage colony-stimulating factor (GM-CSF) and B cell activating factor (BAFF) (Luminex) in the coculture system from GCA. (**C**) Representative OPAL staining of GCA-affected aorta tissue for CD20 (B cells, yellow), CD90 (fibroblasts, green), IL-6, GM-CSF and BAFF (red). Colocalization of two markers is shown in cyan. Arrows indicate double positive cells. (**D**) Masson staining in B cell clusters (blue indicates collagen). (E) Heatmap showing 34 proteins (which passed the quality check in Olink) in the coculture supernatants of activated B cells and fibroblasts, either in direct cell–cell contact or in a transwell system. Protein levels are shown as *Z*-scores and visualized using a red–blue colour scale, where red indicates higher and blue indicates lower expression. Hierarchical clustering was performed using Euclidean distance and complete linkage. Fold change was calculated with fibroblast monocultures as the reference condition. *P < 0.05, **P < 0.01. aB: activated B cells; Adj. *P*-val: adjusted *P*-value; B: B cells; cc: cell–cell contact; F: human aortic adventitial fibroblasts; tr: transwell

### B Cell–fibroblast cell–cell contact may amplify their inflammatory output

As our previous data suggested that cell–cell contact may boost soluble factor responses, we performed Olink proteomic profiling of coculture supernatants to obtain a broader overview of the factors involved. Thirty-four proteins passed the quality check. Overall, the secreted levels of CD80 and granzyme A (GZMA) in the cell–cell contact coculture model were higher compared with those in the transwell model (Supplementary Fig. S8A). Group-specific analysis revealed that in the coculture model including B cells from GCA patients there were increased levels of CD80, IL-6, GZMA, CCL2, CCL7, CXCL10 and CCL20 under cell–cell contact conditions compared with transwell (Fig. 3E), whereas fibroblasts cocultured with B cells from HCs displayed reduced fibroblast growth factor 2 secretion (Supplementary Fig. S8B).

### Profiling soluble mediators secreted by activated B cells reveals association with inflammation

To gain insight into the soluble mediators involved in the B cell–fibroblast crosstalk, supernatants of resting and activated B cells cultured separately were first analysed using a targeted Luminex assay that included six proteins. We found that activated B cells produced higher levels of BAFF, GM-CSF, CXCL8, MMP-3, IFN-γ and TNF-α compared with non-activated B cells (Fig. 4A). Additionally, we performed proteomic profiling using Olink platform for an unbiased comparison of the secretion profiles of activated B cells from GCA patients and HC. Twenty-two proteins passed the quality check. Activated B cells from GCA patients produced higher levels of CXCL8 compared with those from HC (*P* = 0.02), while no significant differences were observed for other factors (Fig. 4B). Amfireguline (AREG, member of the EGF-protein family) and CSF2 (GM-CSF) showed a trend towards statistical significance (*P* = 0.07). Two distinct clusters were identified within the GCA group comprising patients with either high or low *in vitro* B cell cytokine production. However, no significant differences were found between the clusters with respect to ESR, CRP and GCA clinical subtypes (cranial, large-vessel and combined, with or without PMR; Supplementary Fig. S9A). No significant correlations were observed between plasma levels and activated B cell cytokine production for CXCL8, AREG and CSF2 (Supplementary Fig. S9B).

**Figure 4:**
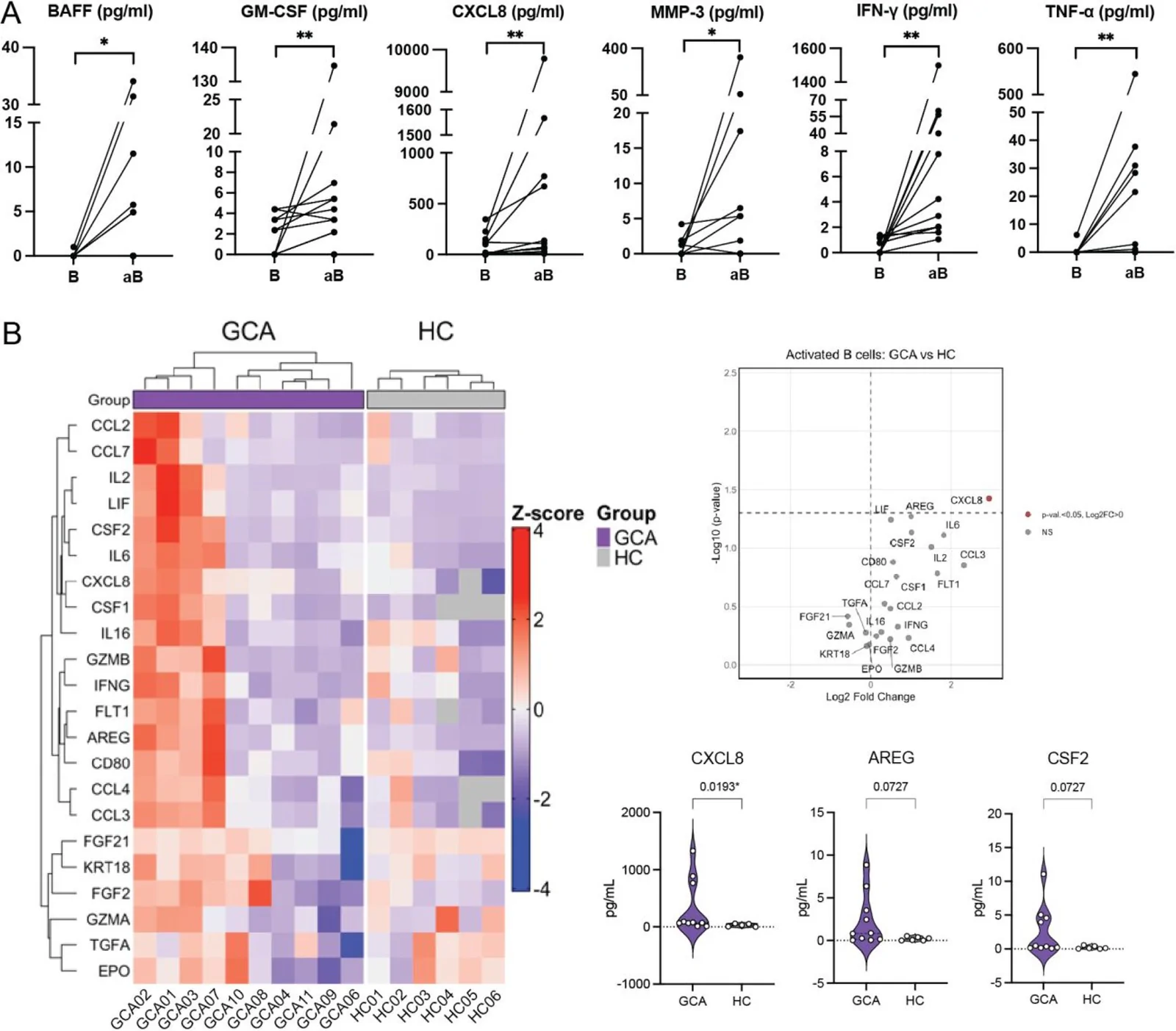
Protein profiles of activated B cells in giant cell arteritis (GCA) and healthy controls (HC). (**A**) Data from a targeted Luminex assay, comparing B cell activating factor (BAFF), granulocyte-macrophages colony stimulating factor (GM-CSF), CXCL8, matrix metalloproteinase (MMP)-3, IFN-γ and TNF-α in the supernatants of resting B cells (B) and activated B cells (aB) from GCA patients. (**B**) Data from Olink, with heatmap representing levels of 22 proteins (which passed the quality check) in the supernatants of activated B cells from GCA (*n* = 10) and HC (*n* = 6). Protein levels are shown as *Z*-scores and visualized using a red–blue colour scale, where red indicates higher and blue indicates lower expression. Hierarchical clustering was performed using Euclidean distance and complete linkage. Volcano plot showing differential protein expression in activated B cells from GCA *vs* HC. Proteins with *P*-value <0.05 and positive log2 fold change are highlighted in red, while non-significant proteins are shown in grey. Here we showed three representative B cells producing cytokines CXCL8, amfireguline (AREG) and GM-CSF(CSF2). *P < 0.05, **P < 0.01

### A Simplified *in vitro* system shows similar effects of activated B cell conditioned medium on fibroblasts as in coculture

We next assessed whether activated B cell conditioned medium (aBCCM) exerts similar effects on HAoAFs as observed in coculture systems, as a simplified *in vitro* model of B cell–fibroblast interaction. Consistent with the pattern observed in the coculture system, exposure to aBCCM from both HC and GCA patients upregulated mRNA expression of *IL-6*, *GM-CSF*, *PDPN* and *FAP* and downregulated *Col1a1* (Supplementary Fig. S6B), but had no significant effect on *BAFF* or *α-SMA* expression in fibroblasts (data not shown). Fibroblasts cultured with aBCCM showed increased IL-6 and reduced pro-collagen Iα1 secretion (Fig. 5A). In the GCA group, IL-6 protein levels correlated inversely with pro-collagen Iα1 levels (Fig. 5B). In line with data from coculture systems, aBCCM from patients with higher CRP levels appeared to induce higher FAP mRNA expression in HAoAF (Supplementary Fig. S6C).

**Figure 5:**
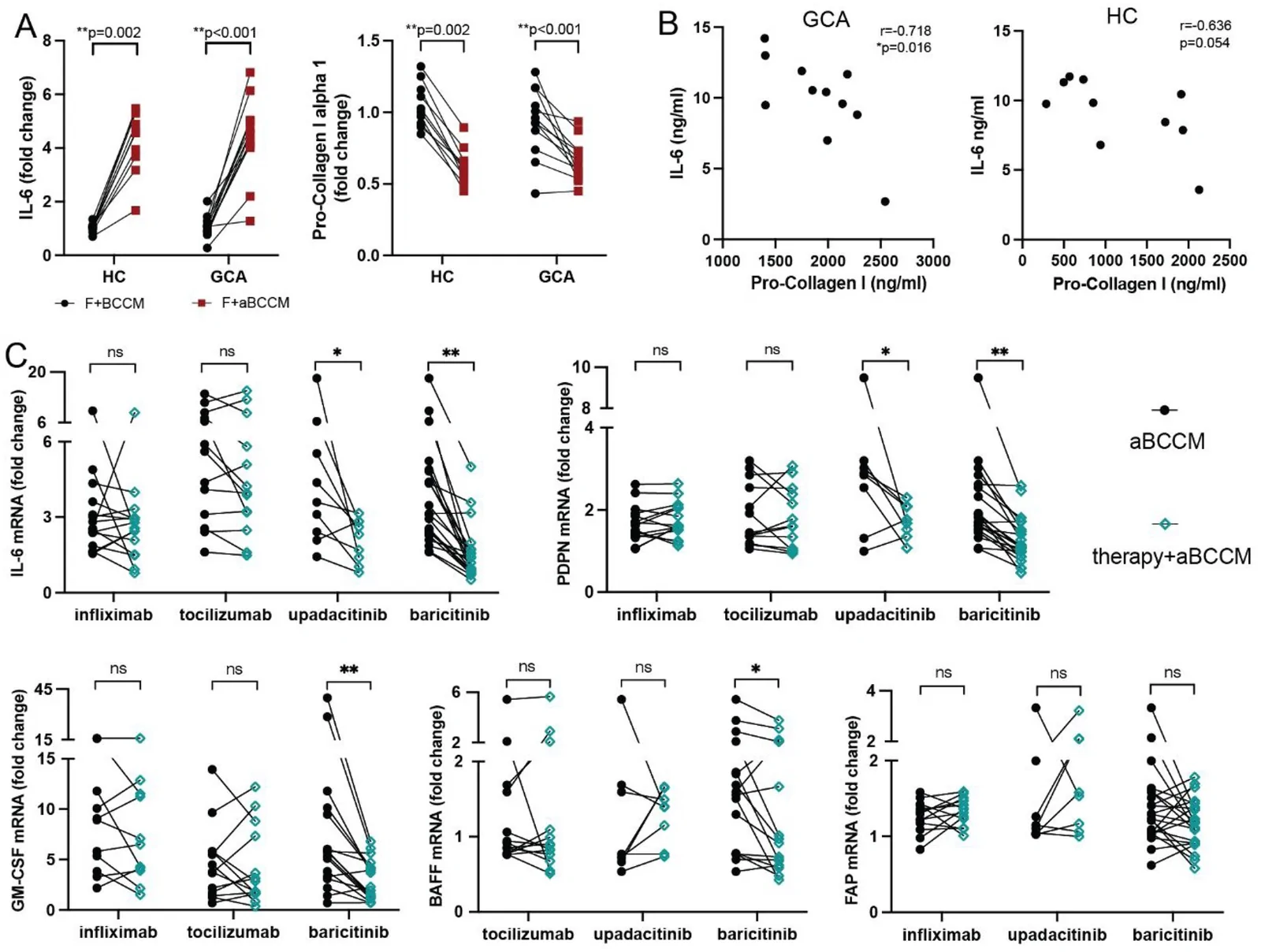
Effects of biologics and Janus kinase–signal transducer and activator of transcription (JAK–STAT) blockers on activated B cell conditioned medium (aBCCM)-induced fibroblast activation. (**A**) Production of IL-6 and pro-collagen Iα1 protein by fibroblasts cultured in aBCCM. Black dots: fibroblasts cultured with BCCM; red squares: fibroblasts cultured with aBCCM. (**B**) Correlation of IL-6 and pro-collagen Iα1 production in fibroblasts when cultured in aBCCM from patients with giant cell arteritis (GCA, left panel) and healthy controls (HC, right panel). (**C**) Effect of biologics (infliximab and tocilizumab) and JAK–STAT blockers (upadacitinib and baricitinib) on fibroblast upregulation of *IL-6*, *podoplanin* (*PDPN*), *granulocyte-macrophage colony-stimulating factor* (*GM-CSF*), *B cell activating factor* (*BAFF*) and *fibroblast activation protein* (*FAP*) mRNA induced by aBCCM. Black dots: fibroblasts cultured with aBCCM; green squares: fibroblasts pretreated with biologicals or JAK–STAT inhibitors for 1 h, followed by culture with aBCCM. *P < 0.05, **P < 0.01; ns, not significant

### JAK–STAT signalling, but not TNF-α signalling or IL-6/IL-6 receptor signalling alone, appears to be involved in B cell-mediated fibroblast activation in GCA

To further explore the signalling pathways involved in the effects of B cells on fibroblasts, we first examined the effects of TNF-α and IL-6 on fibroblasts. TNF-α stimulation significantly increased IL-6 PDPN, GM-CSF and BAFF mRNA expression, whereas FAP mRNA expression was unchanged; in contrast, IL-6 stimulation had no significant effect on the expression of these genes (Supplementary Fig. S10A). We then studied the impact of the TNF-α inhibitor infliximab, the IL-6 receptor (IL-6R) blocker tocilizumab, and the JAK–STAT blockers upadacitinib and baricitinib on aBCCM-exposed fibroblasts. These medications had no significant effect on the viability and morphology of the fibroblasts (Supplementary Fig. S10C and E). Treatment of fibroblasts with infliximab or tocilizumab had no effect on aBCCM-upregulated mRNA expression of *IL-6*, *GM-CSF*, *PDPN*, *BAFF* or *FAP* in these fibroblasts. In contrast, upadacitinib significantly downregulated mRNA levels of *IL-6* and *PDPN* in fibroblasts, whereas the changes in BAFF were not significant. Baricitinib attenuated the aBCCM-induced upregulation of *IL-6*, *GM-CSF*, *PDPN* and *BAFF* mRNA expression in fibroblasts. No significant effect of baricitinib and upadacitinib was found on *FAP* mRNA levels (Fig. 5C).

## Discussion

In this study, we document the presence of distinct fibroblast subtypes (CD90^+^PDPN^+^ imunofibroblasts, CD90^+^FAP^+^ activated fibroblasts, CD90^+^CD248^+^ pro-fibrotic fibroblasts) at the site of B cell clusters within aortic adventitia of patients with GCA. Activated B cells from both GCA patients and HCs skew fibroblasts towards a pro-inflammatory phenotype *in vitro*, which is mainly characterized by elevated expression of IL-6 and GM-CSF and reduced levels of type I collagen. B cells of patients with GCA are more potent inducers of GM-CSF than those of healthy donors. B cell-induced changes in adventitial fibroblasts *in vitro* appear independent of TNF-α and involve JAK–STAT signalling pathways distinct from the IL-6/IL-6R–JAK1/2–STAT3 axis.

We identified a distinct spatial organization of fibroblast subtypes within B cell clusters, suggesting functional specialization of the stromal compartment. In Sjögren’s disease, PDPN^+^FAP^+^ immunofibroblasts drive TLO formation and local pathology by recruiting lymphocytes [15]. Likewise, the central localization of CD90^+^PDPN^+^ immunofibroblasts within aortic B cell clusters may provide a stromal niche supporting B cell recruitment, survival and organization potentially through IL-6 and BAFF production. In contrast, CD248^+^ fibroblasts were located at the cluster periphery. Given the established role of CD248 in tissue remodelling, angiogenesis and fibrosis [16], these fibroblasts, together with neighbouring myofibroblasts, may contribute to ECM remodelling while remaining scarce within clusters to allow B cell nesting. Although FAP is a hallmark of fibroblast activation and is highly expressed across all layers of GCA temporal artery and predominantly in the disrupted media of GCA aorta [13], its expression within clusters was variable and generally limited. We previously demonstrated that adventitial FAP^+^ fibroblasts from human aortas proliferate in inflamed arteries and *in vitro*, and express IL-6 and MMP-9 within GCA vascular lesions [11, 13]. Whether these activated fibroblasts directly contribute to TLO formation in GCA remains to be elucidated. Notably, the overall abundance of fibroblast markers (CD90, FAP, PDPN, CD248, α-SMA) did not differ significantly between GCA and atherosclerosis aortas, suggesting that fibroblasts constitute a common stromal niche supporting adventitial B cell clusters in both conditions. Thus, disease-specific features observed in GCA are likely driven by differences in immune interactions rather than variations in fibroblast abundance. This concept is supported by studies in Takayasu arteritis [17], which have reported reduced B cell–fibroblast communication compared with HCs, indicating that qualitative differences in stromal-immune interactions, rather than stromal cell composition alone, may distinguish pathogenic mechanisms across vascular inflammatory diseases.

*In vitro*, activated B cells skewed fibroblasts toward a proinflammatory phenotype, mainly characterized by enhanced IL-6 and GM-CSF production. Similar observations have been reported in rheumatoid arthritis (RA), where activated or age-associated B cells promoted IL-6 production by fibroblasts-like synoviocytes [18, 19]. While this effect occurs largely independently of cell contact in RA, our findings suggest that in GCA soluble mediators are sufficient to induce fibroblast-derived IL-6 and GM-CSF, with direct cell–cell interactions further amplifying this response. Importantly, fibroblasts cocultured with activated B cells from patients with GCA produced higher levels of IL-6 and GM-CSF than those cocultured with activated B cells from HCs, indicating that GCA B cells may possess an enhanced capacity to promote pro-inflammatory fibroblast responses. Whether this reflects an intrinsic functional alteration of the entire B cell compartment or the expansion of specific B cell subsets remains unknown. Recent studies have highlighted substantial heterogeneity among tissue-infiltrating B cells, including activated, memory, age-associated and plasma cell-like populations with distinct immunoregulatory properties. Future studies integrating high-dimensional B cell phenotyping with coculture assays will be important to delineate subset-specific mechanisms underlying B cell–fibroblast interactions in GCA.

We showed that inhibiting JAK1/2–STAT signalling with baricitinib or upadacitinib, but not blocking TNF-α or IL-6/IL-6R signalling alone, largely reversed the pro-inflammatory effects of B cells on fibroblasts. Although TNF-α is abundantly expressed in GCA tissues [14] and promotes IL-6 production in human aortic fibroblasts [11], clinical studies with TNF-α blockers failed as GC-sparing therapies in GCA [20]. The IL-6R antagonist tocilizumab is clinically effective in GCA, yet its effects were shown to be stronger in PBMCs than in GCA tissue (temporal artery and aorta), where it reduces STAT3 phosphorylation and STAT3-dependent molecules, such as SOCS3, CCL-2 and ICAM-1 [21]. The JAK–STAT pathway integrates downstream signalling from a broad range of cytokines and growth factors. Tofacitinib, which primarily targets JAK1/JAK3, disrupted adventitial microvascular angiogenesis, reduced outgrowth of hyperplastic intima, and suppressed immune responses in human GCA arteries engrafted into immunodeficient-mice [22]. Baricitinib, which primarily inhibits JAK1/JAK2, was both safe and effective in patients with relapsing GCA in an open-label study [23]. Upadacitinib, which predominantly targets JAK1, was recently approved for GCA treatment following the SELECT-GCA trial [3]. In line with these clinical observations, we found that pharmacological inhibition of JAK signalling in aortic fibroblasts markedly attenuated B cell-induced pro-inflammatory activation. Pretreatment of aortic fibroblasts with baricitinib or upadacitinib significantly attenuated activated B cell-induced responses, reducing IL-6 and PDPN mRNA expression. Additionally, baricitinib suppressed GM-CSF and BAFF mRNA expression. Noteworthy, neither upadacitinib nor baricitinib reduced FAP mRNA expression, likely reflecting its regulation through alternative upstream pathways such as TGF-β signaling [11]. Emerging strategies targeting TGF-β and stromal mechanosensing, as successfully applied in cardiac fibrosis, may therefore be employed to limit pathological remodelling in GCA [24].

Among the candidate mediators identified in this study, IFN-γ and CXCL8 represent biologically plausible regulators of fibroblast activation. Previous studies have shown that IFN-γ and TNF synergistically activate fibroblasts and induce the production of CXCL9, CXCL10 and CXCL11 [25]. IFN-γ activates fibroblasts via JAK1/2-STAT1 signalling, inducing MHC class I/II expression, chemokine production and antigen-presenting capabilities [26]. CXCL8 was significantly elevated in activated B cells from GCA patients. Beyond its role as a neutrophil chemoattractant, CXCL8 has potent angiogenic properties and is also markedly upregulated in abdominal aortic aneurysm [27]. This increase may reflect a disease-specific functional skewing of B cells in GCA. A balanced CXCL8/CD248 axis may contribute to the pro-angiogenic environment required for neocapillary and neolymphatic formation in and around TLOs. Future studies dissecting the effects of individual cytokines in isolation, as well as their downstream signalling and cellular mechanisms, will be required to identify the specific mediators driving fibroblast transcriptional reprogramming.

The strength of this study lies in its combined descriptive and mechanistic approach, supported by extensive *in vitro* experiments, to elucidate how B cells from treatment-naïve GCA patients influence adventitial fibroblasts. The use of an *in vitro* system also allowed us to study the effects of targeted therapies on adventitial fibroblasts. A limitation of this study is the relatively small sample size of the tissue and *in vitro* analyses, underscoring the need for validation in larger, independent cohorts to confirm and extend our findings. Another limitation is the use of commercially available primary human aortic adventitial fibroblasts derived from a single donor, which does not capture inter-donor stromal heterogeneity. Future studies incorporating primary fibroblasts from multiple donors will be important to validate and extend these findings.

In conclusion, our study provides further support for an antibody-independent role of B cells in GCA, which may contribute to disease chronicity and progression by inducing and maintaining a pro-inflammatory fibroblast phenotype at the site of vasculitis. Targeting B cell and fibroblast crosstalk may represent a promising approach to modulate both inflammation and vascular remodelling in GCA. Furthermore, this study provides additional evidence supporting the role of JAK–STAT inhibition in treating vascular inflammation in GCA.

## Supplementary Material

keag563_Supplementary_Data

## Data Availability

Data are available upon reasonable request to the corresponding author.
