# Supplementary material for: Activated B cells steer fibroblasts towards a proinflammatory phenotype in giant cell arteritis
Source: Rheumatology (Oxford). 2026 Oct 19;65(10):keag563. doi: 10.1093/rheumatology/keag563 (PMC13707385; doi:10.1093/rheumatology/keag563)
Supplement: Supplementary file 1 [file keag563_supplementary_data.zip › rhe-26-1373-File003.docx]

**Supplementary Data S1. Supplementary methods**

**Patients and controls**

To study B cells and fibroblasts in tissues, aorta tissue samples from patients with histologically-proven GCA (n=9) and age- and sex-matched patients with atherosclerosis (n=11) who underwent aortic aneurysm surgery were included. Patients’ clinical and laboratory data at the time of surgery were extracted from their medical records. All procedures were in compliance with the Declaration of Helsinki. Consent from the Internal Review Board and written patient consent were not required under the Dutch law for human medical research (WMO) since this was a retrospective, non-interventional study (UMCG Research Register number: 201800370). Patients were informed that obtained medical data could be used for research purposes in accordance with the General Data Protection Regulation of the European Union.

The *in vitro* part of the study was performed with data and samples collected in a prospective longitudinal observational cohort that includes patients with GCA, the associated disease polymyalgia rheumatica (PMR) and healthy elderly (SENEX), named GPS. The SENEX cohort comprises healthy individuals older than 50 years of age recruited from the general population of the northern part of the Netherlands. At inclusion, participants had no relevant past and present diseases and did not take any immunosuppressive drugs. Peripheral blood mononuclear cells (PBMC) from 12 treatment-naïve patients with GCA and 10 age- and sex-matched healthy donors were used for B cell isolation. All study participants provided written informed consent before inclusion. The study was approved by the institutional review board of the University Medical Center Groningen (METc2010/222).

**Immunohistochemistry (IHC)**

Paraffin-embedded aorta sections were deparaffinized in xylene and rehydrated with graded ethanol, followed by antigen retrieval in a heated Tris-EDTA buffer (pH=9) bath. The tissue sections were then incubated with primary antibodies targeting CD20, CD90, α-SMA, FAP, PDPN, and CD248, followed by secondary antibodies, 3,3’-Diaminobenzidine (DAB) and hematoxylin (Supplementary Table S1). Semi-quantitative scoring was performed using a 5-point scale: 0= no positive cells, 1= occasional positive (0-1%), 2= a small number of positive cells (>1-20%), 3= a moderate number of positive cells (>20-50%), 4= a large number of positive cells (>50%). Areas containing at least 50 aggregated B cells and with a CD20 score>3 was defined as a B cell cluster (Supplementary Figure S1). The scores of all B cell clusters within each aorta were quantified and averaged. Scores were evaluated by 2 independent investigators. In cases of disagreement, QuPath-based analysis was introduced to support consensus scoring.

**OPAL multiplex immunofluorescence workflow**

Paraffin-embedded aorta sections were deparaffinized in xylene and rehydrated with graded ethanol, followed by antigen retrieval in a heated Tris-EDTA buffer (pH=9) bath. The tissues were subsequently incubated with primary antibody, secondary HRP antibody, and OPAL. After washing with demi water, the cycle was repeated twice starting from antigen retrieval, and continuing with incubation with different primary antibodies, secondary antibodies and OPAL[1]. The tissues were then incubated with DAPI for 10 min. Image cubes were captured at a magnification of 200× by Nuance Multispectral Imaging System 3.0.1 (PerkinElmer, Waltham, MA, USA) using NuanceFX 3.0.1 software (PerkinElmer) (440:460 for DAPI=blue, 490:530 for OPAL480=green, 570:600 for OPAL570=red, 710:720 for OPAL650=yellow). Quantification of overlapping areas was evaluated by NuanceFX 3.0.1 software. The patterns of OPAL staining are shown in Supplementary Table S2.

**PBMC isolation and B cell isolation**

PBMCs were isolated from peripheral blood by density gradient centrifugation with LymphoPrep (STEMCELL^TM^ Technologies) and frozen in RPMI medium with 10% fetal calf serum (FCS) and 10% dimethylsulfoxide (DMSO). B cells were isolated from PBMCs through negative selection magnetic separation using the EasySep^TM^ Human B Cell Isolation Kit (STEMCELL^TM^ Technologies). After isolation, part of the cells was labelled with fluorescently conjugated antibodies targeting surface markers CD19 (Per Cp Cy5.5), CD22 (PE), and CD38 (Pe-Cyanine7) to check purity. The labelled cells were then fixed with FACS fixation buffer (BioLegend) to preserve the cells’ characteristics. The samples were measured on a FACSymphonyTM analyzer (BD Biosciences) and data were analyzed using Kaluza software (BD). B cell enrichment led to a purity of over 90%, as determined by flow cytometry (Supplementary Figure S2).

B cells at a concentration of 400,000 cells/ml in RPMI+10%FCS+0.3% Gentamycin were stimulated with CpG oligodeoxynucleotides (ODN) 2006 (Hycult Biotech) for 67 hours, followed by adding phorbol 12-myristate 13-acetate (PMA; Sigma) and calcium ionophore (CaI; Sigma) for 5 hours[2]. Cells were then washed twice, and subsequently incubated in RPMI+10%FCS+0.3%Gentamycin (100/μl) to allow cytokine production for 24 hours, or used for coculture directly. Control groups were included to evaluate the impact of leftover CpG+PMA+CaI after being washed twice on fibroblast morphology and heterogeneity (Supplementary Figure S10B).

**Fibroblast culture**

Human aortic adventitial fibroblasts (HAoAF) were obtained from PromoCell (C-12380), originating from the thoracic aorta of a 50-year-old non-smoking Caucasian male donor known with hypertension and no inflammatory disease. Cells were routinely cultured in low glucose DMEM supplemented with 10% FCS, 50 μg/ml gentamycin, 10 μg/ml insulin-transferrin-selenium-sodium pyruvate (ITS-A), 1 ng/ml basic fibroblast growth factor (bFGF) and 500 μM Vit-C and were grown at 37 °C in a 5% CO_2_ humidified chamber. Cells used for all experiments were between passages 4 and 8. Passages occurred every 2-3 days, when cells reached 90%-100% confluency.

**Coculture of B cells and fibroblasts**

HAoAFs were seeded onto 24-well plates at a density of 25.000 cells/cm^2^ and reached 50% confluency after 24 hours. Stimulated or unstimulated B cells (10.000/well) were added to HAoAF with/without transwell and incubated for 24 hours. Groups of HAoAFs only, B cells only, and activated B cells only were set as controls. Cell supernatants were collected to detect protein secretion, and HAoAF were harvested for RNA isolation. A detailed coculture protocol is shown in Supplementary Figure S3.

**Fibroblasts cultured in B cell conditioned medium (BCCM)**

Activated B cells were cultured in RPMI medium at the concentration of 100.000/ml for 24 hours, and the resulting cell supernatant was collected as BCCM. HAoAFs were seeded onto 24-well plates at a density of 25.000/cm^2^ and reached 50% confluency after 24 hours. BCCM from stimulated or unstimulated B cells were added to HAoAF and incubated for 24 hours. The ratio of BCCM to fibroblast culture medium was 1:1.

To investigate the possible role of cytokine production and signaling involved in B cell-fibroblasts crosstalk, fibroblasts were left untreated or treated with 10 ng/ml TNF-α inhibitor infliximab (MCE, HY-P9970), 50 nM JAK-STAT blocker baricitinib (MCE, HY-15315), 1µM JAK-STAT blocker upadacitinib (HY-19569) or 9 µg/ml IL-6 receptor blocker tocilizumab (Roche) for 1 hour. BCCM from activated B cells was then added to the fibroblasts without removing these biologicals. As control, HAoAFs were stimulated with TNF-α (2, 20 or 200 ng/mL) or IL-6 (1, 10, or 100 ng/mL). After 24 hours, fibroblasts were collected for RNA isolation (Supplementary Figure S4). The chosen dosage of biologicals was based on literature[3-6] and company information.

**Real-time quantitative PCR (qPCR)**

Total RNA was isolated from fibroblasts using TRIzol, and complementary DNA (cDNA) was obtained by reverse transcription using the PrimeScript RT Master Mix Kit (Invitrogen). qPCR was performed using the Absolute QPCR ROX mix on a ViiA7 machine (Applied Biosystems). Relative standard curve method (2^-ΔCT^) was used to determine the relative mRNA expression with probes targeting *GAPDH* (Hs99999905_m1), *IL-6* (Hs00174131_m1), granulocyte macrophage colony-stimulating factor (*GM-CSF*) (Hs00929873_m1), *PDPN* (Hs00366766_m1), *FAP* (Hs00990791_m1), *α-SMA* (Hs00909449_m1), *CD20* (Hs00544819_m1), *MMP-9* (Hs00957562_m1), *CXCL9* (Hs00171065_m1), *CXCL13* (Hs00968305_m1), *BAFF* (Hs00198106_m1), *Col1a1* (Hs00164004_m1). 2^-ΔΔCT^ was used to compare the fold change in gene expression between the treated and control groups. Gene expression was normalized to that of GAPDH.

**Enzyme-linked immunosorbent assay (ELISA), Luminex assay, and Olink**

Cell supernatants were analyzed using Human IL-6 DuoSet ELISA (DY206, R&D systems ™) and Human Pro-Collagen I alpha 1 DuoSet ELISA (DY6220-05) kits according to the manufacturer’s instructions. 40-fold diluted samples and 5000-fold diluted samples were measured in duplicate using a BioTek EPOCH 2 microplate reader for IL-6 and Pro-Collagen I alpha 1, respectively. The mean of the two measurements was calculated (standard curve range 0-600 pg/ml for IL-6, 0-2000 pg/ml for pro-collagen I alpha 1).

Levels of CXCL8, TNF-α, IFN-γ, B cell activating factor (BAFF), GM-CSF, and MMP-3 were determined using the Human premix Magnetic Luminex screening assay ki t (R&D Systems, Abingdon, UK) and read on a Luminex Magpix instrument (Luminex, Austin, TX, USA).

In addition, 89 proteins were measured in monoculture and coculture supernatants using the Olink Target 48 Cytokine Panel and Olink Target 48 Immune Surveillance Panel (Uppsala, Sweden). All samples were randomized and plated prior to measurement, and then processed in Signature Q100 Sequencing Systems (Arcadia, Utrecht, the Netherlands). Proteins with >20% missing values or below the limit of detection in >80% of samples were excluded from downstream analyses.

References

1.	Taube JM, Roman K, Engle EL, Wang C, Ballesteros-Merino C, Jensen SM, et al. Multi-institutional TSA-amplified Multiplexed Immunofluorescence Reproducibility Evaluation (MITRE) Study. J Immunother Cancer 2021; 9(7).

2.	Graver JC, Jiemy WF, Altulea DHA, van Sleen Y, Xu S, van der Geest KSM, et al. Cytokine producing B-cells and their capability to polarize macrophages in giant cell arteritis. J Autoimmun 2023; 140:103111.

3.	Corbera-Bellalta M, Kamberovic F, Alba-Rovira R, Planas-Rigol E, Prieto-Gonzalez S, Farran-Centelles N, et al. The IL-6 axis in vascular inflammation: effects of IL-6 receptor blockade on vascular lesions from patients with giant-cell arteritis. Ann Rheum Dis 2025; 84(8):1387-1400.

4.	Crivelli SM, Quadri Z, Vekaria HJ, Zhu Z, Tripathi P, Elsherbini A, et al. Inhibition of acid sphingomyelinase reduces reactive astrocyte secretion of mitotoxic extracellular vesicles and improves Alzheimer's disease pathology in the 5xFAD mouse. Acta Neuropathol Commun 2023; 11(1):135.

5.	Wei YF, Li X, Zhao MR, Liu S, Min L, Zhu ST, et al. Helicobacter pylori disrupts gastric mucosal homeostasis by stimulating macrophages to secrete CCL3. Cell Commun Signal 2024; 22(1):263.

6.	Fridman JS, Scherle PA, Collins R, Burn TC, Li Y, Li J, et al. Selective inhibition of JAK1 and JAK2 is efficacious in rodent models of arthritis: preclinical characterization of INCB028050. J Immunol 2010; 184(9):5298-5307.
