# Supplementary material for: Activated B cells steer fibroblasts towards a proinflammatory phenotype in giant cell arteritis
Source: Rheumatology (Oxford). 2026 Oct 19;65(10):keag563. doi: 10.1093/rheumatology/keag563 (PMC13707385; doi:10.1093/rheumatology/keag563)
Supplement: Supplementary file 1 [file keag563_supplementary_data.zip › rhe-26-1373-File004.docx]

**Supplementary Tables**

**Supplementary Table S1. Antibodies used for immunohistochemistry (IHC) staining**

| Target | Antigen retrieval | Manufacturer & code | Host & isotype | Dilution | Secondary antibody | Dilution |
| --- | --- | --- | --- | --- | --- | --- |
| FAP | pH 9 | abcam ab207178 | Rabbit mono IgG | 1:150 | R&D VC003 | undiluted |
| CD90 | pH 9 | NOVUS NBP2-37330 | Mouse IgG1 | 1:400 | DAKO Envision K4001 | undiluted |
| PDPN | pH 9 | DAKO M361929-2 | Mouse IgG1 | 1:40 | DAKO Envision K4001 | undiluted |
| CD248 | pH 9 | Invitrogen MA5-26838 | Mouse IgG1 | 1:75 | DAKO Envision K4001 | undiluted |
| αSMA | pH 9 | abcam ab124964 | Rabbit mono IgG | 1:1000 | DAKO P448 | 1:50 |
| CD20 | pH 9 | DAKO M0755 | Mouse IgG2a | 1:50 | DAKO P260 | 1:50 |
| BAFF | pH6 | NOVUS NBP1-97622 | Rat mono IgG | 1:300 | abcam ab97057 | 1:500 |
| GM-CSF | pH9 | Neobiotechnologies 1437-MSM3-P1ABX | Mouse IgG2c | 1:1000 | DAKO Envision K4001 | undiluted |

**Supplementary Table S2. OPAL staining patterns**

|  | OPAL520 (green) | OPAL570 (red) | OPAL650 (yellow) | DAPI |
| --- | --- | --- | --- | --- |
| CD90/CD20/PDPN | CD90 | PDPN | CD20 | Nucleus |
| CD90/CD20/CD248 | CD90 | CD248 | CD20 | Nucleus |
| CD90/CD20/FAP | CD90 | FAP | CD20 | Nucleus |
| CD90/CD20/IL-6 (sc-130326) | CD90 | IL-6 | CD20 | Nucleus |
| CD90/CD20/BAFF | CD90 | BAFF | CD20 | Nucleus |
| CD90/CD20/GM-CSF | CD90 | GM-CSF | CD20 | Nucleus |

* Diluent: CD90 1:300; CD20 1:50; CD248 1:75 (overnight); FAP 1:100 (overnight); IL-6 1:50; BAFF 1:300; GM-CSF 1:1000.

**Supplementary Figures**

**Supplementary Figure S1. Illustrative examples of scores for fibroblast markers in B cell clusters**. 0 = no positive cells, 1 = occasional positive (0-1%), 2 = a small number of positive cells (>1-20%), 3 = a moderate number of positive cells (>20-50%), 4 = a large number of positive cells (>50%).

**Supplementary Figure S2. B cell enrichment through negative selection magnetic separation using the EasySep^TM^ Human B Cell Isolation Kit**

**Supplementary Figure S3. Detailed protocol for B cell-fibroblast cocultures.** PMA = phorbol 12-myristate 13-acetate, CpG = CpG oligodeoxynucleotides (ODN) 2006, CaI = calcium ionophore.

**Supplementary Figure S4. Schematic overview illustrating the collection of B cell conditioned medium (BCCM) from (activated) B cells and subsequent exposure of fibroblasts to BCCM.** PMA = phorbol 12-myristate 13-acetate, CpG = CpG oligodeoxynucleotides (ODN) 2006, CaI = calcium ionophore.

**Supplementary Figure S5. Immunohistochemical staining of fibroblast markers in GCA-affected aorta tissues.** (A) Overview of expression of CD20, CD90, PDPN, FAP, CD248 and α-SMA in GCA-affected aorta tissues. (B) Representative high magnification images of CD20, CD90, FAP, CD248, PDPN, α-SMA in B cell clusters. CD = cluster of differentiation; PDPN = podoplanin; FAP = fibroblast activation protein; SMA = smooth muscle actin; GCA = giant cell arteritis.

**Supplementary Figure S6. Additional data on coculture systems and activated B cell conditioned medium (aBCCM)-induced fibroblast activation**. (A) Relative mRNA expression (relative to GAPDH) in fibroblast-B cell cocultures. (B) Effects of aBCCM on fibroblasts. (C) Correlation matrix of CRP, ESR and mRNA results. IL-6 = interleukin-6, GM-CSF = granulocyte macrophage-colony stimulating factor, PDPN = podoplanin, FAP = fibroblast activation protein alpha, α-SMA=alpha smooth muscle actin, Col1a1=type I collagen alpha 1. F = fibroblasts, B = B cells, aB = activated B cells, tr = transwell, cc = cell-cell contact. *p<0.05, **p<0.01, ns = not significant.

**Supplementary Figure S7. Additional ELISA and Luminex data showing levels of cytokines and collagen in monoculture and coculture systems.** (A) Dot plot representation of ESR/CRP correlation with FAP, GM-CSF, PDPN and IL-6. (B) IL-6 protein levels in B cells, activated B, fibroblasts or coculture medium. (C) Luminex showing protein levels of B cell activating factor (BAFF), granulocyte macrophage-colony stimulating factor (GM-CSF), C-X-C motif chemokine ligand 8 (CXCL8), matrix metalloproteinase-3 (MMP-3), interferon-γ (IFN-γ), tumor necrosis factor alpha (TNF-α) in different monoculture and coculture systems (GCA group). F = fibroblasts, B = B cells, aB = activated B cells, tr = transwell, cc = cell-cell contact. *p<0.05, **p<0.01, ns = not significant.

**Supplementary Figure S8. Comparison of protein profiles in supernatants of activated B cell-fibroblast cocultures under direct cell contact and transwell conditions.** (A) All, (B) healthy controls (HC). Heatmap showing protein expression levels in the coculture supernatants of activated B cells and fibroblasts, either in direct cell contact or in a transwell system. Protein levels are shown as Z-scores and visualized using a red-blue color scale, where red indicates higher and blue indicates lower expression. Hierarchical clustering was performed using Euclidean distance and complete linkage.

**Supplementary Figure S9. Clinical characteristics and plasma cytokine profiles in relation to B-cell cytokine production.** (A) Clinical features of patients in 2 clusters. Cluster 1 comprised patients whose B cells exhibited higher cytokine production, and cluster 2 comprised patients with lower B cell cytokine production. No clear association was observed between the clusters and ESR, CRP, GCA clinical subtypes (cranial, large-vessel, or combined) or the presence of PMR. (B) Plasma cytokine levels of CXCL8, AREG, CSF2 and their association with activated B-cell cytokine production. *p<0.05, **p<0.01, ns = not significant.

**Supplementary Figure S10. Effects of B cell activation and immunomodulatory drugs on fibroblast morphology, viability, and cytokine expression in co-culture system.** (A) Effect of TNF-α and IL-6 on mRNA expression of FAP, IL-6, PDPN, GM-CSF and BAFF in human aortic adventitial fibroblasts. (B) Effect of PMA+CpG+CaI on fibroblast morphology and mRNA expression of IL-6, GM-CSF, PDPN. 0.01X or 1% = 1:100 dilution of PMA+CpG+CaI used for B cell activation, 0.1X=1:10 dilution, 0.01%=1:10000 dilution. 0.1X and 0.01X showed no cytotoxicity. (C&E) Infliximab, baricitinib, tocilizumab and upadacitinib had no significant impact on viability and morphology of fibroblasts. (D) Controls for TNF-α and infliximab. F=fibroblasts, B = B-cells, aB = activated B-cells, tr = transwell, cc=cell-cell contact. *p<0.05, **p<0.01, ns = not significant.
